# Nonacog beta pegol prophylaxis in children with hemophilia B: safety, efficacy, and neurodevelopmental outcomes for up to 8 years

**DOI:** 10.1016/j.rpth.2024.102341

**Published:** 2024-02-08

**Authors:** Karin S. Walsh, Christine Mrakotsky, Manuel Carcao, Anthony K.C. Chan, Pernille Højlund Nielsen, Helle Holst, Kevin Shapiro

**Affiliations:** 1 Center for Neuroscience and Behavioral Medicine, Children’s National Hospital and the George Washington University School of Medicine and Health Sciences, Washington, DC, USA; 2 Departments of Neurology & Psychiatry, Boston Children’s Hospital, Harvard Medical School, Boston, Massachusetts, USA; 3 Division of Haematology/Oncology, Department of Paediatrics, the Hospital for Sick Children, Toronto, Ontario, Canada; 4 Department of Paediatrics, McMaster Centre for Transfusion Research, McMaster Children’s Hospital, McMaster University, Hamilton, Ontario, Canada; 5 Novo Nordisk A/S, Copenhagen, Denmark; 6 Cortica Healthcare and Children’s Hospital Los Angeles, Westlake Village, California, USA

**Keywords:** bleed, children, hemophilia B, nonacog beta pegol, PEG

## Abstract

**Background:**

Nonacog beta pegol (N9-GP) is an extended half-life PEGylated factor (F)IX product with established efficacy and short-term safety in persons with hemophilia B (HB). Long-term safety has been evaluated for polyethylene glycol exposure but not N9-GP.

**Objectives:**

To assess safety, neurodevelopmental, and efficacy outcomes of children with HB receiving N9-GP prophylaxis across 2 open-label, single-arm, phase 3 studies: paradigm5 (previously treated patients [PTPs]) and paradigm6 (previously untreated patients [PUPs]) in this interim analysis.

**Methods:**

PTPs (aged ≤12 years) and PUPs (aged <6 years) with severe/moderate (≤2% FIX level) HB were recruited to N9-GP prophylaxis (40 IU/kg once weekly) in paradigm5 and paradigm6, respectively. Safety assessments included FIX inhibitor incidence, adverse events, neurocognitive and neurologic outcomes, polyethylene glycol concentration in plasma, and medical events of special interest. Efficacy endpoints included bleeds, N9-GP hemostatic effect, and FIX consumption.

**Results:**

Overall, 25 patients in paradigm5 and 50 patients in paradigm6 received N9-GP and were followed for up to 8 and 6 years, respectively. No inhibitory antibodies were reported in PTPs; 4 of the 50 PUPs developed inhibitors. Extensive evaluation revealed no neurocognitive or neurologic concerns with N9-GP use in children during the study period. Across both studies, few adverse events were reported as possibly related to N9-GP. High hemostatic response rate, high treatment adherence, low annualized bleeding rates, and no new target joints were reported.

**Conclusion:**

These data provide the longest follow-up for an extended half-life FIX and confirm the long-term efficacy of N9-GP prophylaxis in children with HB with no observed neurocognitive or neurologic safety concerns.

## Essentials

• Long-term safety data for clotting factor concentrates joined to polyethylene glycol molecules are limited.
• Paradigm 5/6 assesses the long-term safety and efficacy of nonacog beta pegol in children with hemophilia B.
• Safety data showed no impact of long-term nonacog beta pegol on neurologic or neurocognitive development.
• Efficacy results were robust, with high treatment adherence and low bleeding rates.

## Graphical abstract

**Figure:**
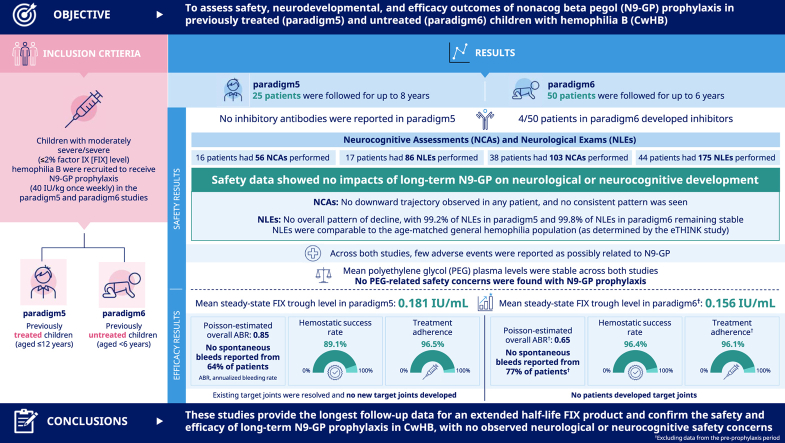

## 1 Introduction

Many persons with hemophilia B experience spontaneous bleeds, particularly in joints and muscles. Debilitating outcomes, such as intracranial hemorrhages (ICH), are more frequent in children with hemophilia B (CwHB) and may result in seizures, psychomotor dysfunction, cognitive impairment, and death [[1], [2], [3], [4]].

For persons with hemophilia B, early prophylactic treatment with coagulation factor concentrates protects them from bleeding and is thus considered the standard of care [5]. Coagulation factor concentrates have been available in clinical practice since the 1970s [6]. However, these products have short half-lives (approximately <30 hours in adults and fewer in children), necessitating frequent injections for prophylactic use [7]. The advent of extended half-life (EHL) factor (F)IX products, with an approximately 3- to 5-fold greater half-life than standard half-life FIX concentrates [8], may benefit persons with hemophilia B by reducing the prophylaxis burden and maintaining efficacious FIX activity levels for longer and with fewer doses vs standard half-life FIX products [9,10].

Nonacog beta pegol (N9-GP; Refixia/Rebinyn; Novo Nordisk A/S) is an EHL product consisting of recombinant FIX with a 40 kDa polyethylene glycol (PEG) moiety attached to the FIX activation peptide [10]. Half-life extension is achieved through this site-specific glycoPEGylation, which preserves FIX enzymatic properties. The pharmacokinetic profile of N9-GP has been extensively characterized [[10], [11], [12], [13]]. When administered as prophylaxis at the approved dosing (40 IU/kg), peak concentration after each dose is high, with mean FIX trough levels of up to 0.273 IU/mL [[10], [11], [12], [13], [14]] and sustained FIX levels >40% for 5.4 d/wk [15,16]. Thus far, the safety and efficacy of N9-GP have been demonstrated through the paradigm clinical program results in previously treated patients (PTPs) and previously untreated patients (PUPs) with hemophilia B [10,11,17]. The ongoing paradigm5 and paradigm6 studies aim to evaluate the long-term safety and efficacy of N9-GP in previously treated (≤12 years of age) or untreated (<6 years of age) CwHB, respectively [18,19].

Animal studies of PEGylated biologics investigating the presence of PEG in the choroid plexus have engendered questions regarding potential adverse neurodevelopmental sequelae in juveniles [10,[20], [21], [22], [23], [24]]. However, a preclinical neurotoxicity study conducted in rats to evaluate the effects of N9-GP on fertility, sexual maturation, and neurodevelopment showed no evidence that PEG exposure adversely impacts neurodevelopment [25].

Establishing the effects of hemophilia on neurodevelopment has historically been challenging, and comparing age-related performance changes against static baseline measures would not be sufficient. To meet this need for a contemporary re-evaluation of the effects of hemophilia on neurodevelopment, the cross-sectional Evolving Treatment of Hemophilia’s Impact on Neurodevelopment, Intelligence, and Other Cognitive Functions study (eTHINK; NCT03660774 [26]) was designed. This pivotal study provided crucial hemophilia-specific age reference norms for cognitive and behavioral outcomes, which are used as measures in the paradigm studies.

Paradigm5 (NCT01467427) and paradigm6 (NCT02141074) are investigating the long-term safety of N9-GP in CwHB through the implementation of extensive clinical safety evaluations, including full narratives of the central nervous system (CNS)–related adverse events (AEs), regular neurologic examinations (NLEs), and neurocognitive assessments (NCAs). Interim reports have been published for paradigm5 and paradigm6, with data cutoffs on October 1, 2018, and August 31, 2018, respectively [11,12]. Here, we report novel results from the interim analysis (data cutoff: November 25, 2020) of paradigm5 and paradigm6 assessing the long-term efficacy and safety (with a focus on neurologic and neurocognitive safety) of N9-GP in CwHB.

## 2 Methods

### 2.1 Study design

**Figure 1:**
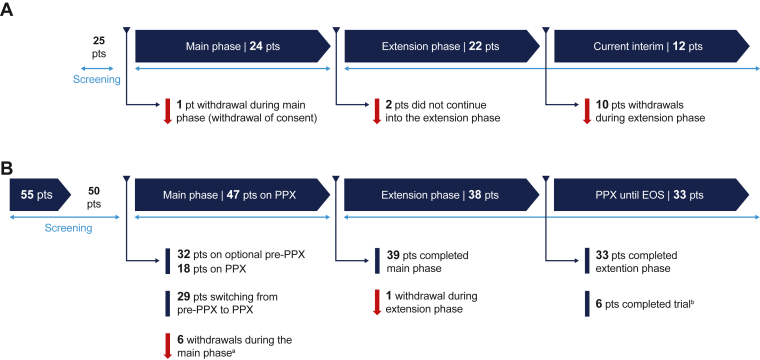
Study design for the (A) paradigm5 and (B) paradigm6 clinical studies. EOS, end of study; PPX, prophylaxis; pt, patient. ^a^Includes 1 patient withdrawn after completing the main phase before entering the extension phase. ^b^Six patients completed the study with follow-up for 24 weeks (±4 weeks apart) after the end of the extension phase; 1 of these patients discontinued treatment after completing the extension phase and is counted as a completer. Withdrawal criteria include the following: incapacity or unwillingness to follow trial procedures, noncompliance, withdrawal of consent, parental concern over PEGylated product, patient discontinuation due to adverse events, discontinuation of the principal investigator at the site, lost patient contact, patient relocation, and trial product not approved for prophylaxis.

paradigm5 and paradigm6 are multinational, open-label, single-arm phase 3 studies conducted in accordance with the Declaration of Helsinki and ICH Good Clinical Practice [11,12]. The designs of both studies are summarized in Figure 1.

paradigm5 investigations were conducted at Novo Nordisk–approved investigational sites in the following countries: Canada, Germany, Italy, Japan, Malaysia, Taiwan, the United Kingdom, and the United States. *Paradigm6 investigations were conducted at Novo Nordisk–approved investigational sites in the following countries: Australia, Austria, Canada, Israel, Japan, Malaysia, Spain, Taiwan, Thailand, the United Kingdom, and the United States*.

Paradigm 5 enrolled PTPs (*N* = 25) ≤12 years of age with ≥50 exposure days (EDs) to any FIX product. The study commenced on May 16, 2012, with completion expected in November 2023. It comprises a 52-week main phase and an ongoing extension phase, which aims to collect data over a 10-year follow-up period [12,17].

Paradigm 6 enrolled PUPs (*N* = 50) <6 years of age with ≤3 EDs to any FIX product. The study commenced on July 2, 2014, with completion in October 2022. The main phase included an optional preprophylaxis period for on-demand treatment of bleeds. Prophylaxis had to be initiated before the patient reached 24 months of age or had received 20 EDs. The main phase was completed after 50 EDs were received, typically within 1 to 3 years of study. Patients could then choose to continue on a 1-year extension phase, possibly remaining in the study until global completion [11].

In both studies, patients received N9-GP 40 IU/kg intravenously once weekly as prophylaxis. Mild/moderate bleeds were treated with 1 dose of N9-GP 40 IU/kg, and trauma/severe bleeds were treated with N9-GP 80 IU/kg.

Patients in both studies were males with severe/moderately severe congenital hemophilia B (FIX activity level of ≤2%). Key exclusion criteria for both studies were: (1) a known history or current presence of FIX inhibitors and (2) any investigational medicinal product received within 30 days before screening.

### 2.2 Objectives, endpoints, and assessments of paradigm5 and paradigm6

#### 2.2.1 Study objectives

The primary objective of both studies was to evaluate the immunogenicity of N9-GP as measured by the incidence of anti-FIX inhibitory antibodies (≥0.6 Bethesda Units). Secondary objectives included evaluation of the safety and efficacy of N9-GP in long-term prophylaxis and treatment of bleeds.

#### 2.2.2 Secondary endpoints and assessments: safety

Secondary endpoints for safety included the number and frequency of AEs (serious AEs [SAEs] and medical events of special interest [MESI]). Various safety assessments, including analysis of biochemistry parameters, PEG plasma sampling, NCAs (assessing potential cognitive impact), and NLEs (assessing potential neurologic impact), were implemented after the study started pursuant to protocol amendments in both studies (December 2017 and June 2018). Although safety data were collected throughout the study for a broad range of AEs, including CNS and neurologic events, the MESI designation allowed for detailed narratives on AEs that could potentially be related to long-term PEG exposure in the same manner as SAE reporting, regardless of severity.

For all patients, NCAs were conducted twice yearly, with primary objectives defined as cognitive and behavioral functions sensitive to change over time, including performance-based attention, processing speed, working memory (Cogstate battery), and self/parent-reported executive function (Behavior Rating Inventory of Executive Function [BRIEF], age-specific versions). For patients in English-speaking countries only, general developmental/intellectual ability was obtained with standardized measures used in routine clinical care, including the Bayley Scales of Infant and Toddler Development and the Wechsler Preschool and Primary Scale of Intelligence/Wechsler Abbreviated Scale of Intelligence. Behavioral status and emotional adjustment were measured via parent and self-reported ratings on the Behavior Assessment System for Children, and adaptive skills were measured with parent ratings and self-reported ratings for adults with the Adaptive Behavior Assessment System (Supplementary Table S1). An individual patient’s performance in each assessment was compared with age-matched reference norms from a CwHB (eTHINK) sample, allowing for age-specific comparisons over time. eTHINK enrolled 551 patients aged 1 to 21 years with any severity of hemophilia A or B, with/without inhibitors, and regardless of treatment experience [26,27].

NLEs assessed 8 domains: general development, cranial nerves, coordination, fine motor skills, reflexes, sensory skills, gait, strength, and tone. These were categorized as “normal” or “abnormal” (preexisting or new). Training was provided to site-level investigators to ensure standardization.

All NCA and NLE results were evaluated by an independent External Expert Review Panel, which included pediatric neuropsychologists, a pediatric neurologist, and a pediatric hematologist every 6 to 12 months on a case-by-case basis. The panel considered various developmental, medical, family history, and psychosocial factors that may have influenced the outcome and whether these factors were study-related.

#### 2.2.3 Secondary objective, endpoints, and assessments: efficacy

Further secondary endpoints included the number of bleeds during prophylaxis (assessed through annualized bleeding rates [ABRs]), the hemostatic effect of N9-GP for treatment of bleeds (estimated using a 4-point categorical scale; “excellent” and “good” were categorized as successful), and peak/trough levels of FIX activity.

#### 2.2.4 Statistical analysis

The European Medicines Agency guidelines were used for the sample size of both studies [11,12,28].

CIs, based on binomial distribution, were used to report the incidence of anti-FIX inhibitory antibodies.

NCA trajectories over time were analyzed through graphical heatmaps of mean change in z-scores from the initial to subsequent assessments, where “green” represented an improvement and “orange” represented a decline in z-score. The z-score represents the number of SDs a domain score deviates from the mean of the normative data for children and young adults with hemophilia, as identified in the eTHINK study. For these studies, a z-score of −1.5 SDs below the mean was defined as a cutoff to identify potential clinical concerns.

To further analyze the association between z-scores and N9-GP exposure, random regression models were generated, including ED as a fixed effect and patient as a random effect. This analysis was only performed for the executive function and attention/processing speed domains due to limited data for other domains.

Shift plots were used to analyze NLE trajectories over time, evaluating the proportion of NLE items that changed categories between examinations. Results were displayed as shifts, with “shift up” indicating improvement and “shift down” indicating worsening. No change was defined as “abnormal” to “abnormal” or “normal” to “normal” findings.

ABRs were modeled using Poisson regression models, allowing for overdispersion and using treatment duration as offset. FIX trough activity measurements were analyzed using a mixed model on the log-transformed plasma concentrations with patients as a random effect. All other endpoints were based on descriptive analyses.

## 3 Results

### 3.1 Patient demographics

In paradigm5, 25 PTPs were enrolled and exposed to N9-GP; 24 completed the main phase, 22 entered the extension phase, and 12 remained at the time of data cutoff (Figure 1A). Ten patients were withdrawn from the extension phase for reasons including protocol violation, withdrawal of consent, loss of contact, and withdrawal of patient by parent or principal investigator. No patients were withdrawn due to an AE [17]. At study entry, the median age was 7.0 years, 3.0 years for the younger age group (0-6 years), and 10.0 years for the older age group (7-12 years; Supplementary Table S2). In total, 150.87 patient-years involving 7924 EDs were reported in the study. A median treatment period of 7.5 years/patient was recorded.

In paradigm6, 55 PUPs were screened, 50 of whom were exposed to N9-GP; 32 entered the preprophylaxis main phase, and 18 immediately entered the prophylaxis phase. In total, 39 patients completed the main phase, 38 entered the extension phase, 33 completed the extension phase, and 37 were still ongoing at the interim cutoff (Figure 1B). Five patients were withdrawn due to AEs (Table 1). The median age at study entry was 1.0 years (Supplementary Table S2). In total, 142.3 patient-years were reported in the study: 22.1 years in the preprophylaxis period and 120.1 years in the prophylaxis period, accounting for 6709 EDs. A median treatment period of 2.7 years/patient was recorded.

**Table 1:** Incidence of adverse events.

	Paradigm5 (150.87 patient-y)				Paradigm6 (142.28 patient-y)			
	*N*^a^	%^b^	E^c^	R^d^	*N*^a^	%^b^	E^c^	R^d^
All AEs	24	96.0	621	4.12	50	100.0	741	5.21
Serious AEs^e^	6	24.0	7	0.05	23	46.0	40	0.28
Fatal^f^	0	0	0	0	1	2	1	0.01
Withdrawal	0	0	0	0	5^g^	10.0	6	0.04
AEs by severity								
Severe	2	8.0	3	0.02	15	30.0	21	0.15
Moderate	15	60.0	60	0.40	28	56.0	80	0.56
Mild	24	96.0	558	3.70	47	94.0	640	4.50
AEs by relationship								
Probably or possibly related^h^	4	16.0	8	0.05	10	20.0	20	0.14
MESIs								
MESI^i^	5	20.0	8	0.05	23	46.0	37	0.26
CNS-related MESIs	2	8.0	2	0.01	11	22.0	17	0.12
Serious CNS-related MESIs	1	4.0	1	0.01	3	6.0	3	0.02

The table does not include linked AEs. For paradigm5, 14 linked AEs (including 3 serious AEs) were reported in 4 patients.

AE, adverse event; CNS, central nervous system; MESI, medical event of special interest.

a Number of patients with AEs.

b Percentage of patients with AEs.

c Number of adverse events.

d Number of AEs per patient-year of exposure (E/total time in trial).

e See Supplementary Text S1 for further details.

f One fatal intracranial hemorrhage event in paradigm 6 is unlikely related to nonacog beta pegol as judged by the investigator.

g Five patients were withdrawn due to AEs: 2 patients with nonsense mutations were withdrawn due to an anaphylactic reaction and/or the development of factor IX inhibitors, 1 patient was withdrawn due to hypersensitivity, 1 patient was withdrawn because of a chronic language disorder due to a suspected delay in cognitive development, and 1 patient was withdrawn due to an intracranial hemorrhage with a fatal outcome.

h As judged by the investigator.

i MESI term updated with CNS.

Full baseline patient characteristics for the paradigm5 and paradigm6 interim analyses are detailed in their respective primary publications [11,12].

### 3.2 Safety results

#### 3.2.1 General safety

In paradigm5, no FIX inhibitors occurred during the study. In paradigm6, 4 of 50 patients developed FIX inhibitors (≥0.6 Bethesda Units; inhibitor incidence rate of 8% [one-sided 97.5% upper CI, 21%]; Supplementary Table S3).

In paradigm5, 24/25 patients reported 621 AEs (4.12 AEs per patient-year of exposure); 7 SAEs were reported in 6 patients, and 8 MESIs (6/8 were allergic reactions; Supplementary Text S1) in 5 patients. Two patients reported CNS-related MESIs, including migraine and Gilles de la Tourette syndrome (Table 1). Furthermore, 4 patients reported 8 events deemed possibly or probably related to N9-GP as judged by the investigator (Supplementary Text S2). In paradigm6, 50 patients reported a total of 741 AEs (5.21 AEs per patient-year of exposure); 23 patients reported 40 SAEs and 37 MESIs (Supplementary Text S1). Of the 37 reported MESIs, 17 were CNS-related; 3 of which were serious and included autism spectrum disorder, language delay, and ICH leading to death (this patient, before his first N9-GP exposure, had suffered from a spontaneous ICH event, and a second ICH while on N9-GP; Table 1). Ten patients reported 20 events deemed possibly or probably related to N9-GP, as judged by the investigator, including 1 instance of language disorder and 1 of autism spectrum disorder (Supplementary Text S2).

In both studies, the mean steady-state PEG plasma concentration was stable over time and ranged from 4.7 to 6.9 μg/mL throughout the trial period. Steady-state concentration was reached at approximately 6 months of continuous prophylaxis.

#### 3.2.2 NCAs and NLEs for paradigm5 and paradigm6

Sixteen patients in paradigm5 and 38 patients in paradigm6 had 56 (mean, 3.5 assessments per patient) and 103 (mean, 2.7 assessments per patient) NCAs performed, respectively.

The random regression models showed no association between N9-GP EDs and NCA composite z-scores for attention performance (Cogstate Attention Composite; *estimate of coefficient [est.]* = −0.0059 and −0.0011) or executive function ratings (Global Executive Composite on parent-reported BRIEF-Preschool/BRIEF2 [parent]; *est.* = −0.0015 and 0.0007), in paradigm5 and paradigm6, respectively. Further, the Global Executive Composite on self-report (BRIEF2-Self and BRIEF-Adult), only administered in paradigm5, also did not demonstrate any significant association with EDs (*est.* = 0.0027; Table 2).

**Table 2:** Trajectory of primary neurocognitive outcomes (z-scores) over time based on random regression models.

Fixed effect	Cogstate battery: Attention Composite							
	Patients, *n*		Assessments, *n*		Est.		*P* value	
	p5	p6	p5	p6	p5	p6	p5	p6
EDs	16	17	53	41	–0.0059	–0.0011	0.106	0.764

Fixed effect	BRIEF-Preschool, BRIEF2: Global Executive Composite (parent report)							
	Patients, *n*		Assessments, *n*		Est.		*P* value	
	p5	p6	p5	p6	p5	p6	p5	p6
EDs	13	35	43	88	–0.0015	0.0007	0.625	0.650

Fixed effect	BRIEF2, BRIEF2-SR, BRIEF-A: Global Executive Composite (self-report)			
	Patients, *n*	Assessments, *n*	Est.	*P* value
	p5	p5	p5	p5
EDs	12	36	0.0027	0.675

Random regression models are shown with ED as a fixed effect and patient as a random effect, allowing for a random intercept and random slope for ED for each patient.

BRIEF, Behavior Rating Inventory of Executive Function; BRIEF2, BRIEF2 parent assessment (age range, 6-17 years); BRIEF2-SR, BRIEF2 self-report (age range, 11-17 years); BRIEF-A, BRIEF adult assessment (age range, 18-90 years); BRIEF-Preschool (age range, 2-5 years); EDs, exposure days; Est., estimate of coefficient; p5, paradigm5; p6, paradigm6.

**Figure 2:**
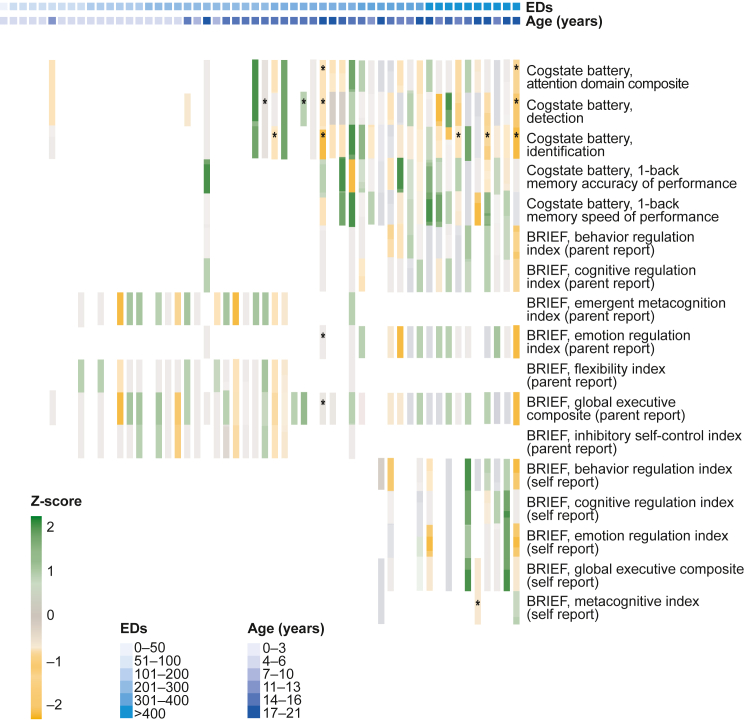
Change in primary outcome measures of neurocognitive assessments for paradigm5 and paradigm6 did not show a consistent pattern of impairment. Mean change in cognitive performance for Cogstate battery and parent or self-reported ratings of executive functions (Behavior Rating Inventory of Executive Function [BRIEF]; BRIEF-Preschool, BRIEF-2, and BRIEF-A) between initial and subsequent assessments by patient age and exposure days (EDs) for paradigm5 and paradigm6^a^ combined. Z-scores compare an individual’s performance against the normative Evolving Treatment of Hemophilia's Impact on Neurodevelopment, Intelligence, and Other Cognitive Functions (eTHINK) study population. If ≥3 neurocognitive assessments (NCAs) were available, then the mean change from initial to each subsequent NCA is included in the heatmap. For each individual patient, orange represents a decrease in z-scores from the initial assessment, and green represents an increase. Patients with only 1 assessment for an instrument are not displayed in the heatmap. An ∗ denotes that the most recent score is less than –1.5 (or 1.5 SDs below the Evolving Treatment of Hemophilia's Impact on Neurodevelopment, Intelligence, and Other Cognitive Functions hemophilia population mean). ^a^Fifty-six NCAs were performed in paradigm5 and 103 in paradigm6.

Heatmaps for the BRIEF and Cogstate measures showed variability within and across patients and instruments for mean change in z-scores against hemophilia age reference norms (eTHINK [26]). The variation in change between assessment scores yielded no consistent pattern of decline or improvement (Figure 2).

Seventeen patients in paradigm5 and 44 in paradigm6 had 86 (mean, 5.1 examinations per patient) and 175 (mean, 4.0 examinations per patient) NLEs performed, respectively. These examinations demonstrated no overall pattern of decline in neurologic status, with 99.2% (paradigm5) and 99.8% (paradigm6) of patients remaining stable.

**Figure 3:**
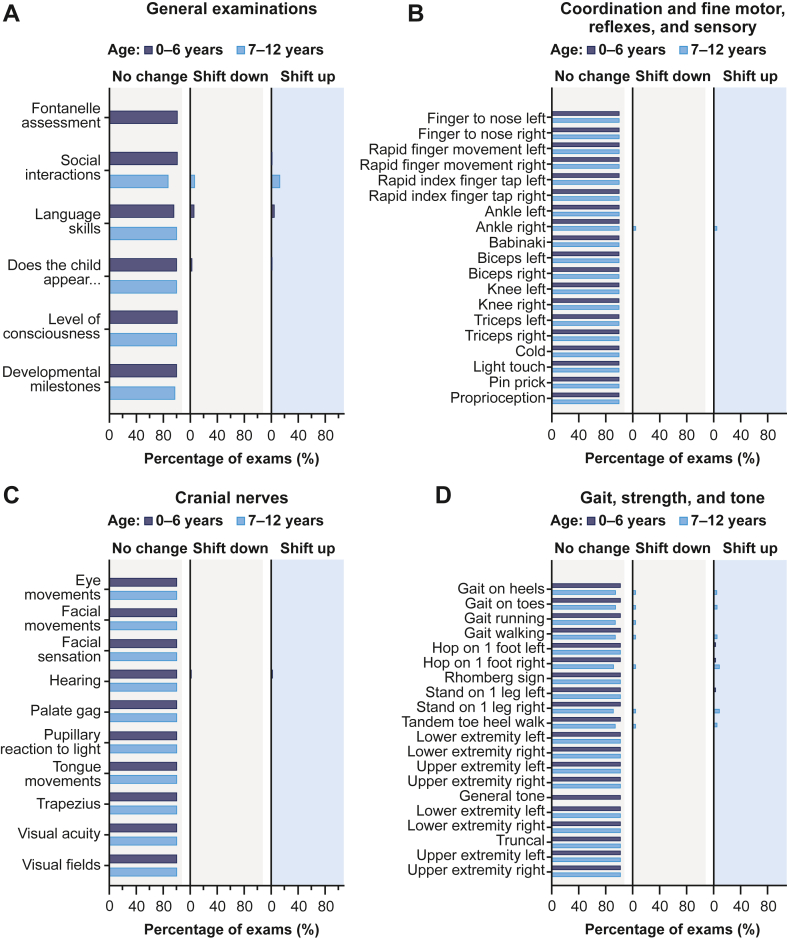
Change in functional domains of neurologic examinations (NLEs) for paradigm5 and paradigm6. NLE shift plots for (A) general examinations, (B) coordination and fine motor skills, reflexes, and sensory skills, (C) cranial nerves, and (D) gait, strength, and tone. Data are displayed from both paradigm5 and paradigm6 studies. NLEs were assessed in terms of shifts up and down. A shift up indicated a patient’s change in skill from “abnormal” to “normal,” and a shift down indicated a change from “normal” to “abnormal” on 2 consecutive evaluations. No change is defined as “normal” to “normal” or “abnormal” to “abnormal.”

For general NLEs in paradigm6, changes over time in language were most frequently reported: 5 patients (13%) reported a shift down, of which 2 (5%) reported a shift up at a subsequent visit (Figure 3A). Overall, there were no significant changes in coordination, fine motor skills, cranial nerves, gait, strength, and tone evaluations (Figure 3B–D). For coordination, fine motor functioning, reflexes, and sensory examinations were normal, except for a transient shift down for 1 patient (7-12 years age group) in paradigm5 (Figure 3B). All examinations were normal for gait, strength, and tone, except for inconsistent findings for gait in 1 paradigm5 patient and 3 abnormal findings for gait in a paradigm6 patient, which normalized in subsequent examinations (Figure 3D).

### 3.3 Efficacy results

**Figure 4:**
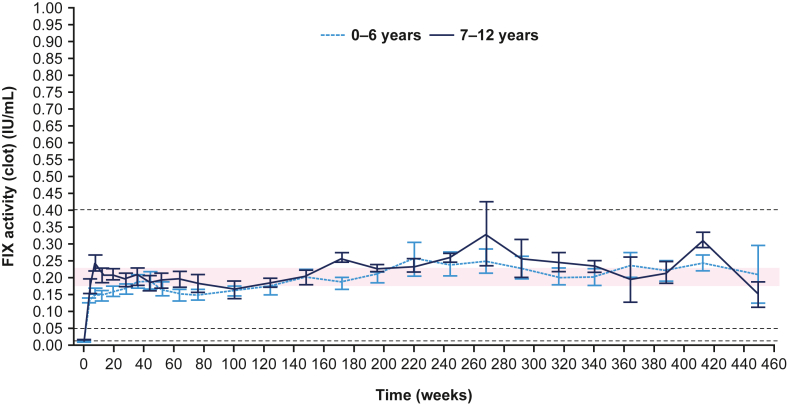
Mean factor (F)IX trough activity levels (IU/mL) in paradigm5 over the study periods for the paradigm5 study comparing children aged 0 to 6 years and 7 to 12 years.

At steady state, mean FIX trough levels were estimated to be 0.181 IU/mL (95% CI, 0.160-0.204) in paradigm5 and 0.156 IU/mL (95% CI, 0.144-0.170) in paradigm6 (Figure 4 and Supplementary Figure S1, respectively).

In paradigm5, with a treatment duration of up to 8 years, 20 (80.0%) patients were treated for 128 bleeds. Five (20%) patients experienced no bleeds during the study, and 16 (64%) experienced no spontaneous bleeds. With a treatment duration of up to 6 years in paradigm6, 35 (70.0%) patients were treated for 148 bleeds. Fifteen (30%) patients experienced no bleeds during the study, and 34 (68%) experienced no spontaneous bleeds.

For paradigm5, all reported bleeds were mild/moderate; 40 (31.3%) were spontaneous, and 84 (65.6%) were traumatic. The Poisson-estimated overall, spontaneous, and traumatic ABR decreased over the analysis period (Supplementary Figure S2A). The Poisson-estimated overall ABR for the entire paradigm5 study period was 0.85. In paradigm6, 141 (95.3%) of the reported bleeds were mild/moderate, 36 (24.3%) were spontaneous, and 109 (73.6%) were traumatic. The Poisson-estimated overall, spontaneous, and traumatic ABRs in the paradigm6 main phase (excluding the preprophylaxis period) similarly decreased over the analysis period (Supplementary Figure S2B). The Poisson-estimated overall ABR for the entire paradigm6 study period was 0.65.

Overall, the hemostatic response success rate when treating bleeds was 89.1% and 96.4% in paradigm5 and paradigm6, respectively. Treatment adherence remained high, with 96.5% and 96.1% adherence (defined as a prophylactic dose administered 6 to 8 days after the last dose) in paradigm5 and paradigm6, respectively.

Finally, in paradigm 5, 2 patients reported 2 existing target joints, which were later resolved. No new target joints were reported in either study.

## 4 Discussion

The studies in this analysis aimed to evaluate the long-term safety and efficacy of N9-GP prophylaxis in CwHB. Patients received N9-GP treatment for a median of 7.5 and 2.7 years in paradigm5 and paradigm6, respectively, and results demonstrated sustained efficacy and no identifiable safety issues.

EHL factor products have demonstrated low inhibitor rates for both PTP and PUPs with hemophilia [[29], [30], [31], [32]]. Similarly, no patients in paradigm5 developed anti-FIX inhibitors. PUPs in paradigm6 reported an 8% incidence of anti-FIX inhibitors, which falls within the expected range (5%-23%) for the PUP population [[33], [34], [35], [36], [37]]. Overall, inhibitor development in severe hemophilia B is rare, as missense variants account for most cases; however, inhibitor development is relatively common in patients with severe disease with null mutations, such as nonsense mutations [34]. In paradigm6, 21% of patients reported nonsense mutations. Of the 4 patients who developed inhibitors, 3 had nonsense mutations, and 1 had a large deletion mutation. Two of these patients had previously been exposed to other recombinant FIX products, and thus, it is unclear whether inhibitor development was a direct result of treatment with N9-GP (Supplementary Table S3) [30,[33], [34], [35], [36], [37]].

Following protocol amendments to expand safety evaluations, N9-GP was well tolerated, with only a few possibly related AEs (as judged by investigators). The majority of CNS-related MESIs were mild/moderate in severity. In paradigm5, 2 patients experienced CNS-related MESIs: migraine and Gilles de la Tourette syndrome, both deemed unlikely related to N9-GP by the investigator as the patient with the migraine recovered soon after experiencing only 1 event, and with the latter, there was a family history of the disorder. Of the reported CNS-related MESIs in paradigm6, the following were reported as serious: autism spectrum disorder, language delay, and a fatal spontaneous ICH. There was no clear relationship between PEG/N9-GP exposure and autism spectrum disorder or language delay in these patients. The patient who died from an ICH after N9-GP prophylaxis initiation had experienced a spontaneous ICH event only a few months prior; thus, it was deemed unlikely to be related to N9-GP by the investigator.

The current analysis suggests that N9-GP exposure is not associated with safety concerns related to the choroid plexus. This is supported by analysis of plasma samples from previous paradigm studies, which demonstrated that systemic PEG levels remain stable during N9-GP prophylaxis for up to 6.5 years after the initial increase in plasma-PEG levels to a steady state (across all ages) [11,12].

Since children and adolescents, particularly when younger, are at critical stages in their development, they could be susceptible to products that affect neurodevelopment, with potential long-term implications; hence, neurologic and neurocognitive outcomes were explored extensively in these studies. NCAs remained stable in the study sample over time and were comparable to the eTHINK hemophilia/age reference population. Similarly, NLEs remained stable over time, although 13% of participants reported language delays, which is only slightly above the incidence observed in the general population (12%) [38].

Across both studies, N9-GP prophylaxis led to high and stable FIX trough values at steady state (0.181 IU/mL and 0.156 IU/mL in paradigm5 and paradigm6, respectively), contributing to low ABRs and high hemostatic success rates. The higher estimated mean FIX trough levels at steady state for paradigm5 may be explained by the patients’ older median age (7.0 years) compared with paradigm6 (1.0 years). Overall, both studies showed an expected and gradual increase in mean trough FIX levels over time; this is expected as FIX clearance decreases with age [39,40].

Finally, there was no target joint development, treatment adherence levels were high, and ABRs were favorable and comparable with or lower than other approved EHL FIX products [41,42]. However, results cannot be directly compared due to differences in study design and patient populations.

### 4.1 Limitations

A limitation of both studies was the small sample size, which limited the generalizability of the results. However, this is common in interventional studies for rare diseases. In addition, patient retention in long-term follow-up studies is challenging and becomes more difficult the longer patients remain. The majority of patients lost from paradigm5 and paradigm6 were due to relocation and care being transferred from a pediatric to an adult care center. Retention was additionally complicated by N9-GP becoming licensed in many jurisdictions and hence being available outside of these studies.

Within NCAs, inconsistencies were observed between the findings from parent-reported and self-reported rating scales, perhaps due to developmental variability, rater perception, and rater reporting style. Nevertheless, combined with objective cognitive assessments, these rating scales are complementary and can provide holistic views of development.

Finally, despite being the longest analysis of neurodevelopmental safety outcomes in CwHB, such clinical investigation does not entirely preclude the possibility of longer-term safety signals. Therefore, the importance of appropriate safety surveillance studies remains.

## 5 Conclusion

In conclusion, the results displayed no neurologic, neurocognitive, or PEG-related safety concerns with N9-GP use for up to 8 years, thus confirming its overall tolerability. The extensive safety, neurodevelopmental, and efficacy outcomes presented here further support the use of once-weekly N9-GP prophylaxis for CwHB and contributed to US Food and Drug Administration approval for a label expansion for routine prophylaxis in adults and children [43].
