# Supplementary figures and images for: Nonacog beta pegol prophylaxis in children with hemophilia B: safety, efficacy, and neurodevelopmental outcomes for up to 8 years
Source: Res Pract Thromb Haemost. 2024 Feb 8;8(2):102341. doi: 10.1016/j.rpth.2024.102341 (PMC10955654; doi:10.1016/j.rpth.2024.102341)

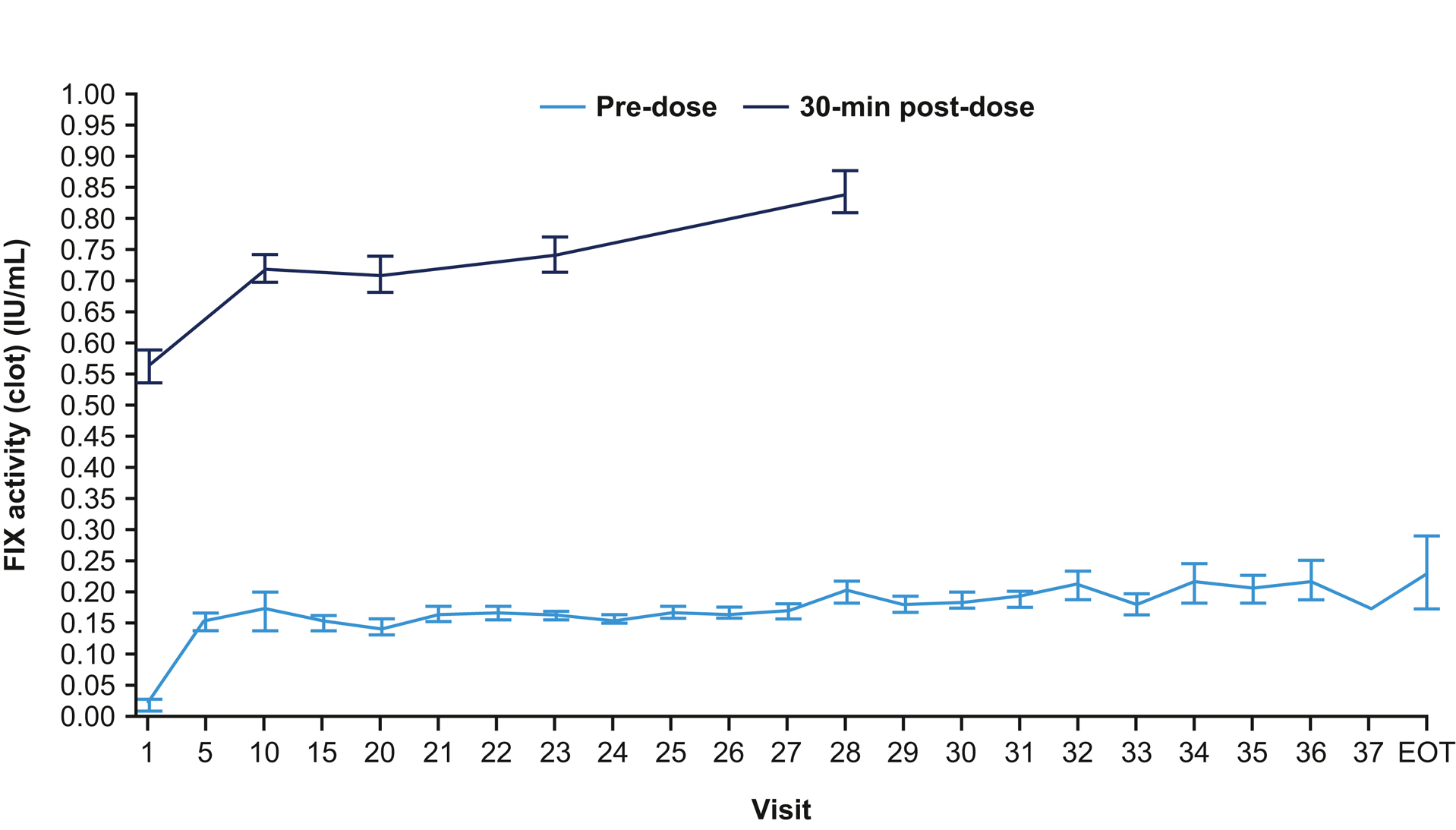

Supplement: Supplementary Figure 1 [file figs1.jpg]

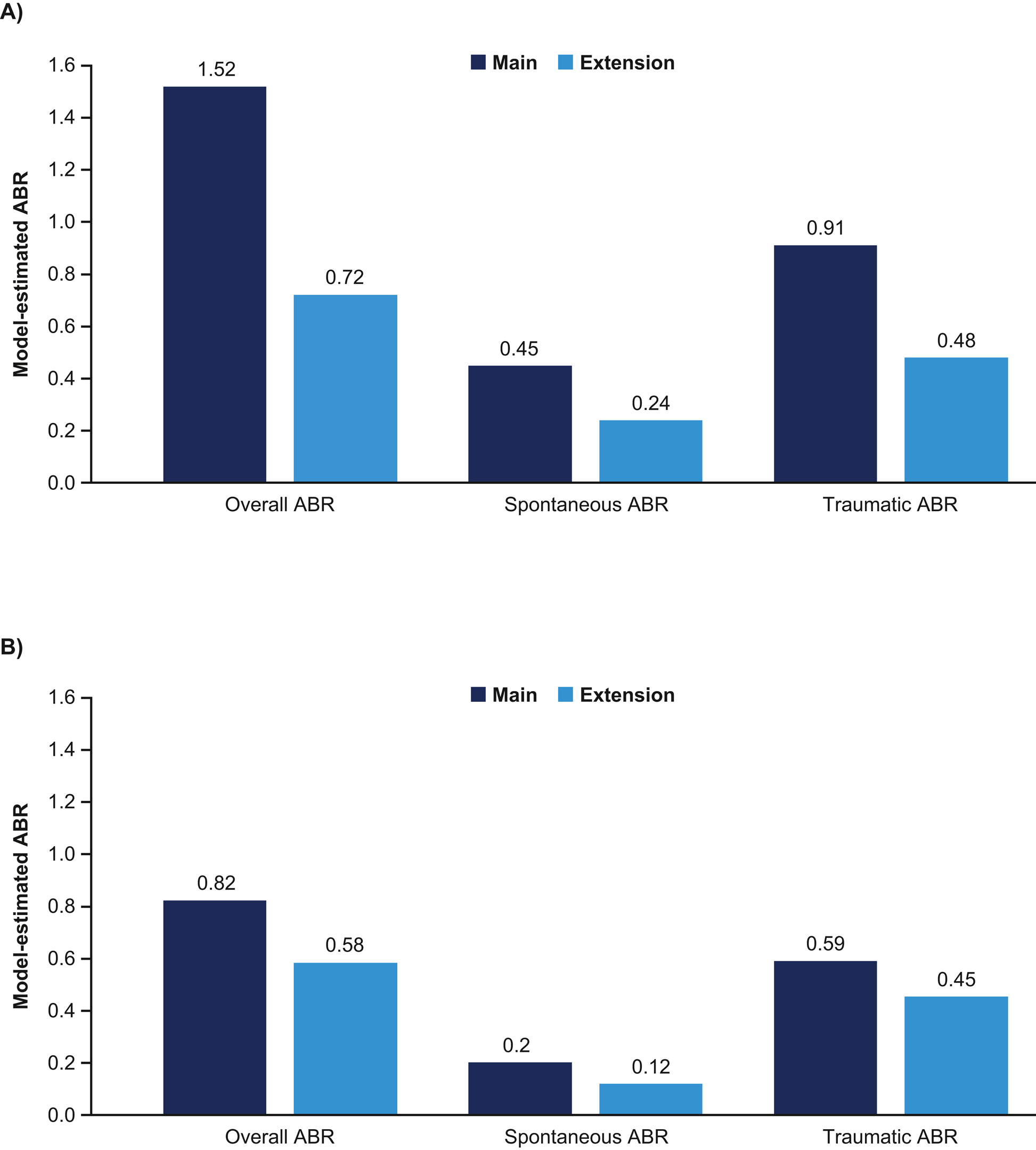

Supplement: Supplementary Figure 2 [file figs2.jpg]
