# Supplementary material for: Nonacog beta pegol prophylaxis in children with hemophilia B: safety, efficacy, and neurodevelopmental outcomes for up to 8 years
Source: Res Pract Thromb Haemost. 2024 Feb 8;8(2):102341. doi: 10.1016/j.rpth.2024.102341 (PMC10955654; doi:10.1016/j.rpth.2024.102341)
Supplement: Supplementary material [file mmc1.docx]

**SUPPLEMENTARY MATERIAL**

**APPENDIX A Serious adverse events reported in the paradigm5 and paradigm6 studies**

In paradigm5, seven SAEs were reported in 6 patients. These included: infections and infestations, Gilles de la Tourette syndrome, food poisoning, radius fracture, and hemoptysis.

In paradigm6, 40 SAEs were reported in 23 patients; 20 SAEs were reported and categorized as infections or infestations, which is expected in a young patient population. Other SAEs included: FIX inhibition, platelet dysfunction, ICH, language disorder, dental caries, anaphylactic reaction, hypersensitivity, fall and head injury, positive blood culture, cardiac catheterization, autism spectrum disorder, hematuria, Henoch-Schönlein purpura, and poor venous access.

**APPENDIX B Adverse events possibly or probably related to the study product**

In paradigm5, a total of eight AEs were reported in 4 patients as possibly or probably related to N9-GP, as judged by the investigator. These AEs included: abdominal pain, diarrhea, nausea, infusion site pain, injection site pain, eosinophilia, headache, and wheezing.

In paradigm6, a total of 20 AEs were reported in 10 patients as possibly or probably related to N9-GP, as judged by the investigator. These AEs included: hypersensitivity, anaphylactic reaction, drug hypersensitivity, FIX inhibition, rash, infusion site extravasation, pyrexia, hypoacusis, conjunctivitis, accidental underdose, language disorder, and autism spectrum disorder.

**SUPPLEMENTARY TABLE S1 Overview of additional neurocognitive assessments and age groups in English-speaking countries (Australia, Canada, the United Kingdom, and the United States).**

These NCAs were analyzed at every second NCA visit and evaluated by the EERP. There was variability within and between patients throughout the studies, and no clear pattern of decline (defined as –1.5 SDs below the mean z-score) was observed.

| **NCA domain assessed:** | **Neurodevelopment/ general intelligence** | **Emotional behavior** | **Adaptive behavior** |
| --- | --- | --- | --- |
| **Patient age (year: months)** |  |  |  |
| 1:0–1:11 | Bayley-III 1:0–3:6y |  | ABAS-3 0-5  (parent)  1:0–5:11y |
| 2:0–3:6 | Bayley-III  1:0–3:6y | BASC-3 PRS-P (parent)  2:0–5:11y | ABAS-3 0-5  (parent)  1:0–5:11y |
| 3:7–3:11 |  | BASC-3 PRS-P (parent)  2:0–5:11y | ABAS-3 0-5  (parent)  1:0–5:11y |
| 4:0–5:11 | WPPSI-IV  4:0–6:11y | BASC-3 PRS-P (parent)  2:0–5:11y | ABAS-3 0-5  (parent)  1:0–5:11y |
| 6:0–6:11 | WPPSI-IV  4:0–6:11y | BASC-3 PRS-C (parent)  6:0–11:11y | ABAS-3 0-5  (parent)  1:0–5:11y  ABAS-3 5-21  (parent)  6:0–21:11y |
| 7:0–7:11 | WASI-II  7:0–21:11y | BASC-3 PRS-C (parent)  6:0–11:11y | ABAS-3 0-5  (parent)  1:0–5:11y  ABAS-3 5-21  (parent)  6:0–21:11y |
| 8:0–11:11 | WASI-II  7:0–21:11y | BASC-3 PRS-C (parent)  12:0–21:11y  BASC-3 SRP-C  (self-report)  8:0–11:11y | ABAS-3 5-21  (parent)  6:0–21:11y |
| 12:0–15:11 | WASI-II  7:0–21:11y | BASC-3 PRS-A  (parent)  12:0–21:11y  BASC-3 SRP-A  (self-report)  12:0–17:11 y | ABAS-3 5-21  (parent)  6:0–21:11y |
| 16:0–17:11 | WASI-II  7:0–21:11y | BASC-3 PRS-A  (parent)  12:0–21:11y  BASC-3 SRP-A  (self-report)  12:0 – 17:11 y | ABAS-3 5-21  (parent)  6:0–21:11y  ABAS-3 16-89  (adult)  16:0–21:11y |
| 18:0–21:11^a^ | WASI-II  7:0–21:11y | BASC-3 PRS-A  (parent)  12:0–21:11y  BASC-3 SRP-College  (self-report)  18:0–21:11y | ABAS-3 5-21  (parent)  6:0–21:11y  ABAS-3 16-89  (adult)  16:0–21:11y |

ABAS-3, Adaptive Behavior Assessment System, Third Edition; A, adolescent; BASC-3, Behavior Assessment System for Children, Third Edition; Bayley-III, The Bayley Scales of Infant and Toddler Development, Third Edition; C, Child; EERP, External Expert Review Panel; NCA, neurocognitive assessment; PRS, parent rating scale; P, pre-school; SD, standard deviation; SR, self-report; SRP, self-rating of personality; WASI-II, Wechsler Abbreviated Scale of Intelligence; WPPSI-IV, Wechsler Preschool and Primary Scale of Intelligence; y, years.

^a^Questionnaires for patients older than 21:11 will be provided when relevant.

## SUPPLEMENTARY TABLE S2 Patient demographics and baseline characteristics.

|  | **paradigm5^a^**  **(N = 25)** | **paradigm6^b^**  **(N = 50)** |
| --- | --- | --- |
| **Age, *years*** | | |
| Median | 7.0 | 1.0 |
| Mean (SD) | 6.5 (3.7) | 0.8 (1.1) |
| **Race, *n* (%)** | | |
| Asian | 8 (32) | 17 (34) |
| Black or African American | 1 (4) | 6 (12) |
| White | 13 (52) | 24 (48) |
| Other | 3 (12) | 3 (6) |
| **FIX mutations at baseline*,* *n* (%)** | | |
| Frameshift | 1 (4) | 0 (0) |
| Missense | 10 (40) | 24 (50) |
| Nonsense | 6 (24) | 10 (20.8) |
| Large deletion | 2 (8) | 1 (2.1) |
| Small deletion | 3 (12) | 1 (2.1) |
| Small duplication | 0 (0) | 1 (2.1) |
| Small insertion | 0 (0) | 1 (2.1) |
| Splice site | 1 (4) | 7 (14.6) |
| No mutation identified or Unknown | 2 (8) | 2 (4.2) |
| Other | 0 (0) | 2 (4.2) |

FIX, factor IX.

^a^Patients were enrolled from centers in: Canada (n *=* 4), Germany (n *=* 1), Italy (n *=* 1), Japan (n *=* 3), Malaysia (n *=* 2), Taiwan (n *=* 2), the United Kingdom (n *=* 3), and the United States (n = 9).
^b^Patients were enrolled from centers in: Australia (n *=* 2), Austria (n *=* 2), Canada (n *=* 1), Israel (n *=* 2), Japan (n *=* 1), Malaysia (n *=* 4), Spain (n *=* 4), Taiwan (n *=* 6), Thailand (n *=* 6), the United Kingdom (n *=* 4), and the United States (n *=* 18).

**SUPPLEMENTARY TABLE S3 Development of anti-FIX inhibitory antibodies
in paradigm6.**

|  | **Age range** | **Mutation type** | **Exposure days** | **Further details** |
| --- | --- | --- | --- | --- |
| **Patient 1^a^** | 19–24-month-old boy | Nonsense mutation  (exon 2 c.[223C>T], p.[Arg75Ter]) | 3 | The patient was in the prophylaxis phase and suffered a moderate allergic reaction to his **second dose** of N9-GP. The patient was hospitalized due to a severe allergic reaction to the **third dose** of N9-GP, and later developed FIX inhibitors (40.2 BU) |
| **Patient 2** | 13–18-month-old boy | Nonsense mutation  (exon 1  c.[83C>A],  p.[Cys>stop codon]) | 4 | Patient entered study in the pre-prophylaxis phase with a negative FIX inhibitor test. He had been previously exposed to FIX before study entry. Post-enrollment antibody test results revealed positive low-titer N9-GP antibodies. Patient had an anaphylactic reaction within 5 minutes of the **fourth dose** and testing showed a high-titer FIX inhibitor (two positive inhibitor tests were confirmed by central laboratory to be 7.8 and 5.1 BU). Patient was treated with systemic steroids and made a full recovery from the anaphylactic reaction and was subsequently withdrawn from the study |
| **Patient 3^a^** | 13–18-month-old boy | Nonsense mutation  (exon 2 c.[223C>T], p.[Arg75Ter]) | 6 | The patient had previously received treatment with another rFIX product before study entry. He displayed numerous complications at the **fourth dose** of N9-GP (given during pre-prophylaxis phase)**.** A high titer inhibitor was first detected after **the fifth dose** of N9-GP; the titer at the local laboratory was 10.5 BU while at the central laboratory it was 4.5 BU. Patient continued in the study, with no further N9-GP doses and the inhibitor titer gradually disappeared within 5 months; however, the patient was withdrawn following a port-insertion surgery that led to the reappearance of the high-titer inhibitor (15.4 BU) |
| **Patient 4** | 13–18-month-old boy | Large deletion mutation | 32 | Patient in the prophylaxis phase demonstrated decreased recovery and shortened half-life of N9-GP. After **20 EDs**, a positive IgG4 for anti-FIX and after 27 EDs a low titer inhibitor  (0.9–1.0 BU) was detected. Treatment was discontinued and the patient started immune tolerance induction |

BU, Bethesda Units; EDs, exposure days; FIX, factor IX; rFIX, recombinant factor IX; IgG4, immunoglobulin G4; N9-GP, nonacog beta pegol.

^a^Patients not related, despite having a similar mutation.

**SUPPLEMENTARY FIGURE S1 Mean FIX trough activity levels (IU/mL) in paradigm6.**

Mean (± SEM) FIX trough activity levels over the study periods for the paradigm6 study, comparing pre- and 30-minute post-dose levels.

FIX, factor IX; IU, international unit; SEM, standard error of the mean.

## SUPPLEMENTARY FIGURE S2 Annualized bleeding rates over time

ABRs for A) paradigm5 and B) paradigm6. The number of bleeds was analyzed based on a Poisson model allowing for over-dispersion and using treatment duration as an offset. Bleeds treated with other FIX products or bypassing agents are also included.

ABR, annualized bleeding rate; FIX, factor IX.

Across the overall, traumatic, and spontaneous bleeding rates, there was a reduced estimated ABR between the main and extension phases.
